# Does Inpatient Palliative Care Facilitate Home-Based Palliative Care Postdischarge? A Retrospective Cohort Study

**DOI:** 10.1089/pmr.2020.0095

**Published:** 2021-02-01

**Authors:** Mudathira Kadu, Luke Mondor, Amy Hsu, Colleen Webber, Michelle Howard, Peter Tanuseputro

**Affiliations:** ^1^ Institute of Health Policy, Management and Evaluation, University of Toronto, Toronto, Ontario, Canada.; ^2^ Institute for Clinical Evaluative Sciences, Toronto, Ontario, Canada.; ^3^ Bruyère Research Institute, Ottawa, Ontario, Canada.; ^4^ Clinical Epidemiology Program, Ottawa Hospital Research Institute, Ottawa Hospital, Ottawa, Ontario, Canada.; ^5^ Department of Family Medicine, McMaster University, Hamilton, Ontario, Canada.; ^6^ Division of Palliative Care, Department of Medicine, University of Ottawa, Ottawa, Ontario, Canada.

**Keywords:** continuity of care, home-based palliative care, inpatient palliative care, palliative care, transitional care

## Abstract

***Introduction:*** Evidence of the impact of inpatient palliative care on receiving home-based palliative care remains limited.

***Objectives:*** The objective of this study was to examine, at a population level, the association between receiving inpatient palliative care and home-based palliative care postdischarge.

***Design:*** We conducted a retrospective cohort study to examine the association between receiving inpatient palliative care and home-based palliative care within 21 days of hospital discharge among decedents in the last six months of life.

***Setting/Subjects:*** We captured all decedents who were discharged alive from an acute care hospital in their last 180 days of life between April 1, 2014, and March 31, 2017, in Ontario, Canada. The index event was the first hospital discharge furthest away from death (i.e., closest to 180 days before death).

***Results:*** Decedents who had inpatient palliative care were significantly more likely to receive home-based palliative care after discharge (80.0% vs. 20.1%; *p* < 0.001). After adjusting for sociodemographic and clinical covariates, the odds of receiving home-based palliative care were 11.3 times higher for those with inpatient palliative care (95% confidence interval [CI]: 9.4–13.5; *p* < 0.001). The strength of the association incrementally decreased as death approached. The odds of receiving home-based palliative care after a hospital discharge 60 days before death were 7.7 times greater for those who received inpatient palliative care (95% CI: 6.0–9.8).

***Conclusion:*** Inpatient palliative care offers a distinct opportunity to improve transitional care between hospital and home, through enhancing access to home-based palliative care.

## Introduction

Inpatient palliative care delivered by specialists has become an important component of care for end-of-life patients in many developed countries.^1^ In Canada, ∼75% of end-of-life patients will be admitted to acute care in the last six months of life, making it an important care setting for this population.^2^ Studies show that inpatient palliative care is associated with reduced burdensome transitions,^3^ reduced health service utilization and costs,^4^ and increased likelihood of death in the preferred place.^5,6^ This is not surprising, as planning for care after discharge to the home setting is a key component of inpatient palliative care delivery.^7^ Discharge referrals from inpatient palliative consultations can initiate patients' entry into home health care programs,^8^ making it a potentially useful bridge between acute and home care.

Home-based palliative care has been found to be effective and cost-effective.^9^ The receipt of home-based palliative care after hospital discharge has been shown to decrease readmissions and overall health care utilization and its associated costs.^9–12^ When home-based palliative care is delivered, patients often receive health and social care from a multidisciplinary team, including trained palliative nurses, physicians, care coordinators, personal support workers, and other allied professionals.^9^ Home-based palliative care teams can improve communication among patients, their caregivers, and primary care physicians about goals of care and initiate discussions about treatment selection in the comfort of their home.^10,13^ Despite this, only one in five individuals who are at their end of life receive home-based palliative care in Canada.^14^

The association between fragmentated care delivery during the transition from hospital to home and poor discharge outcomes has been previously established in the general population.^15,16^ For the end-of-life population, continuation of palliative care through the postdischarge period can be important in ensuring long-term positive patient and caregiver outcomes.^13–16^ Few studies have examined the relationship between inpatient palliative care and postdischarge home-based palliative care, with majority examining the association between inpatient palliative care and increased residential hospice use.^7,17–19^ Although the benefits of inpatient palliative care and home-based palliative care have been separately established, evidence of whether inpatient palliative care impacts the likelihood of receiving home-based palliative care remains limited.

## Methods

### Study design and setting

We conducted a retrospective cohort study examining the association between receiving inpatient palliative care and home-based palliative care within 21 days of hospital discharge among decedents in the last six months of life. We examined residents in Ontario—Canada's largest province with >14 million residents—who were eligible for services under the province's universal health insurance program (i.e., the Ontario Health Insurance Plan [OHIP]). OHIP covers all medically necessary services, including palliative care services provided in community and institutional settings. Although eligibility and assessment for home-based palliative care services are usually standardized, the decision to refer patients to home-based palliative care from the hospitals is often at the physician's discretion. Referrals may also come from nonphysician providers and patients can also self-refer to home-based palliative care. In Ontario, home-based palliative nursing and home-based palliative physician visits are separate services; a patient may receive either or both simultaneously. Home care services can be provided on a short-term or long-stay basis. The latter is when the client is anticipated to need or receives ongoing support for >60 days.

### Data sources

Our study used health administrative data that were linked deterministically. This was done using uniquely encoded identifiers and analyzed at ICES, an independent nonprofit research institute whose legal status under Ontario's health information privacy law allows it to collect and analyze health care and demographic data, without consent, for health system evaluation and improvement. The use of data in this project was authorized under section 45 of Ontario's Personal Health Information Protection Act, which does not require review by a Research Ethics Board. For description of the administrative databases used in this study, please see Supplementary Table S1.

### Subjects

We captured all decedents who were discharged alive from an acute care hospital in their last 180 days of life between April 1, 2014, and March 31, 2017, in Ontario, Canada. The index event was the first hospital discharge furthest away from death (i.e., closest to 180 days before death). We focused on the last 180 days of life because the eligibility criterion for receiving home care nursing with palliative intent is often an expected death within six months.^20^ Discharges in the last 30 days were not evaluated as they represented a more downstream stage of the palliative care trajectory, punctuated by increased acute care use.^2^ Furthermore, given that we were interested in the receipt of home-based palliative care 21-day postdischarge, we wanted sufficient follow-up time for examining our outcome of interest. Decedents were excluded if they were younger than 18 years or older than 105 years at discharge, or if they were not a resident of Ontario. They were also excluded if they were ineligible for coverage under OHIP at any point in the year preceding the index hospital discharge. As we were interested in persons eligible for home-based palliative care services after hospital discharge, we excluded decedents who were admitted or discharged to a long-term care facility (i.e., nursing homes) within the last 180 days of life.

### Measurements

#### Exposure

We determined whether patients were admitted for the main purpose of palliative care during their index hospitalization using the Canadian Institute for Health Information Discharge Abstract Database (CIHI-DAD). This was based on either having a principal diagnosis of palliative care and/or when the main patient service was palliative care. This approach to capturing palliative care delivered in acute care was adapted from previous research examining this topic in the Ontario setting.^14^

#### Outcome

The primary outcome was whether a decedent received home-based palliative care within 21 days of discharge from the index hospitalization. This could include home-based palliative nursing care and/or a home-based palliative physician visit within 21 days. Despite the 5-day provincial benchmark for accessing home care services, patients continue to experience delays between referral and initiation of home care.^21^ Therefore, 21 days was chosen to allow sufficient time between hospital discharge and the event of interest to occur. Given that hospital readmission was a competing event for receiving home-based palliative care, it was our second outcome of interest.

#### Covariates

Baseline variables were determined at the index hospitalization. Sociodemographic variables included age, gender, year of death, and rurality (urban vs. rural residence). Socioeconomic status was measured using neighborhood income quintile.

We determined the presence of 17 chronic conditions. These conditions were selected based on their large economic impact and high prevalence in the general population.^22,23^ and have been used in multiple research studies of multimorbidity in Ontario.^24–27^ These included acute myocardial infarction (AMI), asthma, any cancer, cardiac arrhythmia, chronic coronary syndrome, chronic obstructive pulmonary disorder (COPD), congestive heart failure (CHF), diabetes, hypertension, nonpsychotic mood and anxiety disorders, other mental illnesses, osteoarthritis, osteoporosis, renal failure, rheumatoid arthritis, and stroke (excluding transient ischemic attack). For individuals diagnosed with AMI,^28^ CHF,^29^ asthma,^30^ diabetes,^31^ COPD,^32^ dementia,^33^ and hypertension,^34^ validated case-ascertainment definitions were used. All other conditions were defined based on the presence of any one inpatient hospital diagnostic code (in the CIHI-DAD) or diagnosis codes that are included in two or more outpatient physician billing claims (using the OHIP data). These were identified using relevant ICD-9 and ICD-10 codes. The full set of diagnostic codes used to ascertain comorbid conditions is provided in Supplementary Table S2. Each condition was defined with administrative data from April 1, 2001, onward. The earliest hospital or billing date was used to identify incident cases. From these data, we defined chronic disease burden based on a simple count of prevalent chronic conditions identified at the index hospitalization.

To determine characteristics of the index hospitalization, we examined the total length of stay for the index hospitalization (measured from the admission and discharge date from the CIHI-DAD) and the type of institution the patient was discharged from (teaching vs. nonteaching hospital). We also determined the number of hospital admissions within six months before the index hospitalization date and the number of prior home-based palliative care visits they received.

### Statistical analysis

The distribution of baseline sociodemographic and clinical characteristics and prior health care utilization were described by whether a decedent received or did not receive inpatient palliative care. Frequency counts and percentages were used to summarize categorical variables, whereas means and standard deviations (SDs) were used for continuous variables. To compare baseline differences between groups, chi-square tests and independent sample *t*-test were used.

Because it was important to account for likelihood of different discharge outcomes in our analyses, multinomial logistic model was applied. We used multinomial logistic regression to determine the associations of inpatient palliative care with home-based palliative care and hospital readmission before home-based palliative care. Three outcomes were examined using the multinomial regression, 0, where no event occurred; 1, where the patient received home-based palliative care within 21 days; and 2, where the patient was readmitted within 21 days. Readmission was examined as one of the possible outcomes as it was a competing event for receiving home-based palliative care. Patients could not receive home-based palliative care if they were readmitted. Therefore, it was important for readmission to be included as a discharge outcome. The multinomial logistic regression models produced adjusted odds ratios (OR) and 95% confidence intervals (CIs), with no inpatient palliative care as the reference group. Multivariate modeling was conducted in three stages. First, an unadjusted model was analyzed. In the second model, to examine the effects of controlling demographic and comorbidity level, these variables were adjusted. In the final model, variables based on prior health care utilization were added.

Sensitivity analyses assessed the influence of the timing of inpatient palliative care before death on the odds of receiving home-based palliative care. To do this, we examined hospitalizations occurring 150, 120, 90, and 60 days before death. This was determined by examining the date of the index hospitalization relative to date of death.

We used SAS Enterprise Guide v6.1 (SAS Institute, Inc., Cary, NC) to build the analytical dataset and Stata/MP v15 (StataCorp, College Station, TX) for all analyses.

## Results

After excluding those who did not meet the study criteria or had missing data, 59,007 decedents were included in this study. Of patients hospitalized in 180 to 30 days before death, 1176 (2.0%) received palliative care in the hospital. Counts according to the study exclusion criteria are given in Supplementary Table S3.

Table 1 presents the characteristics of the study population. The mean age of the study population with inpatient palliative care compared with those without was 70.9 years (SD = 13.4) versus 73.2 years (SD = 13.9). Patients with inpatient palliative care were more likely to be women (51.6% vs. 45.7%; *p* < 0.001). They were also more likely to live in rural settings (18.5% vs. 15.1%; *p* < 0.001). Patients with inpatient palliative care had a lower proportion with 5 or more (of 17) comorbidities compared with those without palliative care (34.4% vs. 47.5%). Those with inpatient palliative care had longer inpatient stay in their index hospitalization (15.2 days, SD = 17.4 vs. 9.2 days, SD = 12.4; *p* < 0.001) than those who did not receive inpatient palliative care. They were also more likely to receive care in nonteaching hospitals (75.6% vs. 65.8%; *p* < 0.001). They were more likely to receive home-based palliative care services in the six months before index hospitalization (37.4% vs. 8.0%; *p* < 0.001) and more likely to be seen by a home-based palliative physician (14.9% vs. 2.9%; *p* < 0.001). Patients who received inpatient palliative care also received an average of 19 additional home-based palliative care visits six months before the index hospitalization (23.9 ± 68.1 vs. 4.9 ± 32.1; *p* < 0.001) than those who did not receive inpatient palliative care.

**Table 1.** Profile of Decedents Who Were Discharged Alive after an Acute Care Hospitalization in the Last 180 Days of Life, April 1, 2014, to March 31, 2017, Ontario, Canada

Variable	Overall (*n* = 59,008)	No inpatient palliative care (*n* = 57,832)	Inpatient palliative care (*n* = 1176)	*p*
Sociodemographics				
Age at discharge (years), mean ± SD	73.2 ± 13.9	73.2 ± 13.9	70.9 ± 13.4	<0.001
Women	27,017 (45.8%)	26,410 (45.7%)	607 (51.6%)	<0.001
Income quintile				0.325
1 (lowest)	13,099 (22.2%)	12,834 (22.2%)	265 (22.5%)	
2	12,359 (20.9%)	12,129 (21.0%)	230 (19.6%)	
3	11,519 (19.5%)	11,294 (19.5%)	225 (19.1%)	
4	11,339 (19.2%)	11,087 (19.2%)	252 (21.4%)	
5 (highest)	10,407 (17.6%)	10,206 (17.6%)	201 (17.1%)	
Rural residence	8976 (15.2%)	8759 (15.1%)	217 (18.5%)	0.007
No. of prevalent diagnoses				
0/1	5187 (8.8%)	5072 (8.8%)	115 (9.8%)	<0.001
2	7100 (12.0%)	6908 (11.9%)	192 (16.3%)	
3	9217 (15.6%)	8979 (15.5%)	238 (20.2%)	
4	9654 (16.4%)	9427 (16.3%)	227 (19.3%)	
5+	27,850 (47.2%)	27,446 (47.5%)	404 (34.4%)	
Length of index acute episode (days)				
Mean ± SD	9.3 ± 12.6	9.2 ± 12.4	15.2 ± 17.4	<0.001
Median (IQR)	6.0 (3.0–11.0)	6.0 (3.0–11.0)	10.0 (5.0–19.0)	<0.001
Nonteaching hospital	38,946 (66.0%)	38,057 (65.8%)	889 (75.6%)	<0.001
Teaching hospital	20,062 (34.0%)	19,775 (34.2%)	287 (24.4%)	
Services provided six months prior				
0 prior hospitalizations	43,125 (73.1%)	42,259 (73.1%)	866 (73.6%)	0.893
1 prior hospitalization	10,484 (17.8%)	10,281 (17.8%)	203 (17.3%)	
2+ prior hospitalization	5399 (9.1%)	5292 (9.2%)	107 (9.1%)	
Had 1+ palliative home care services	5038 (8.5%)	4598 (8.0%)	440 (37.4%)	<0.001
Had 1+ palliative home physician services	1842 (3.1%)	1667 (2.9%)	175 (14.9%)	<0.001
Total palliative home care visits	5.2 ± 33.3	4.9 ± 32.1	23.9 ± 68.1	<0.001
Total palliative home physician visits	0.1 ± 0.9	0.1 ± 0.9	0.5 ± 1.7	<0.001

SD, standard deviation.

After the index hospitalization, 21.0% of the decedents received home-based palliative care. Those who had inpatient palliative care were more likely to receive home-based palliative care (80.0% vs. 20.1%; *p* < 0.001). Within 21 days of hospital discharge, they also had lower readmission rates to the hospital (5.4% vs. 21.1%; *p* < 0.001).

Unadjusted and adjusted results from the logistic regression models are presented in Table 2. All three models consistently demonstrated that patients who received inpatient palliative care were more likely to receive home-based palliative care postdischarge. In the unadjusted analyses, the odds of receiving home-based palliative care after discharge were 15.9 times higher for those with inpatient palliative care (OR: 16.8, 95% CI: 14.3–19.8; *p* < 0.001). After adjusting for sociodemographics, morbidity, and year of death, the odds were comparable (OR: 16.3, 95% CI: 13.9–19.2; *p* < 0.001). The strength of the association remained significant but decreased to 12.4 (95% CI: 10.4–14.8; *p* < 0.001) when the model was further adjusted for all the covariates.

**Table 2.** Association between Receiving Inpatient Palliative Care and Palliative Home Care within 21 Days and Readmission among Decedents after an Acute Care Hospitalization between April 1, 2014, to March 31, 2017, in Ontario

Outcome	Unadjusted OR (95% CI)	Model 1 adjusted OR (95% CI)	Model 2 adjusted OR (95% CI)
Palliative home care			
No inpatient palliative care	REF	REF	REF
Received inpatient palliative care	16.82^*^ (14.33–19.75)	16.34^*^ (13.90–19.20)	12.42^*^ (10.42–14.81)

Model 1: adjusts for age, gender, income quintile, rurality, level of multimorbidity, and year of death. Model 2: adjusts for age, gender, income quintile, rurality, level of multimorbidity, year of death, length of hospital stay, number of prior hospitalizations (last six months), prior palliative care home care (last six months), and type of discharging institution (teaching vs. not).

^*^ *p* < 0.05.

CI, confidence interval; OR, odds ratio.

In all three models, there was no significant association between inpatient palliative care and hospital readmission 21 days after the index hospitalization.

**FIG. 1.**
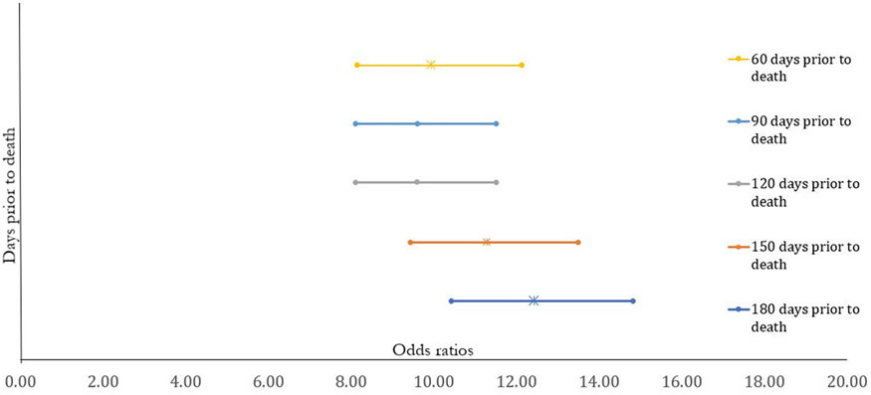
Sensitivity analysis examining the association between the timing of the inpatient care with receiving home-based palliative care.

The sensitivity analysis examining the association between the timing of the inpatient care and receiving home-based palliative care is shown in Figure 1. The closer the timing of receiving inpatient care relative to death, the lower the strength of the association. The strength of the association incrementally decreased between 180 days before death (OR: 11.3, 95% CI: 9.4–13.5) and 60 days before death (OR: 7.7, 95% CI: 6.0–9.8).

## Discussion

This is the first study to establish the association of inpatient palliative care with receiving home-based palliative care postdischarge in Ontario, Canada. Our study found that patients who received inpatient palliative care had greater odds of receiving home-based palliative care within 21 days of discharge. This association was largest when inpatient palliative care was delivered furthest from death. These results are consistent with previous findings that suggest that inpatient palliative care is an effective strategy for anticipating patient needs after discharge, more effective at recognizing these needs at an earlier stage of their illness trajectory and at facilitating referrals and entry into palliative programs after discharge.^8,35–37^

Overall, only 2% of decedents in our sample received inpatient palliative care. However, because we only selected the first index hospitalization event within the last six months of life, this number could underestimate palliative care delivery to recipients, particularly if they experienced multiple hospitalizations before death. Nevertheless, this low proportion of patients receiving inpatient palliative care is noteworthy given the frequent transitions between home and acute care setting experienced by end-of-life patients and the role inpatient palliative care can play in bridging this gap.

Our population-based analysis of decedents showed that 79% of those who received inpatient palliative care also received home-based palliative care, and inpatient palliative care increased these odds by 11 times. This is higher than a prior study by Brody et al.^38^ that demonstrated that 13% of decedents who received inpatient palliative care were discharged to home health care in a large urban nonprofit multicampus hospital in the United States. Brody et al. showed that inpatient palliative care increased the odds by 1.6 times.^38^ The differences in the study designs and care settings could partially explain these findings. Although we examined a population-based cohort of decedents hospitalized in Ontario, Brody et al. examined patients classified as at risk of dying, in a single hospital in the United States.^38^ Furthermore, in Ontario, Canada, although the provincial health insurance plan provides coverage for medically necessary palliative services across all sectors (including home-based care and hospices), end of life is nevertheless often concentrated in hospital inpatient units.^14^ Evidence from the United States shows that patients receiving inpatient palliative care are likely to be discharged to hospices, which could be residential in nature.^5,38–44^

It should be noted that decedents who received inpatient palliative care versus those who did not were more likely to have had home-based palliative care (37.4% vs. 8.0%) and home-based palliative physician services (14.9% vs. 2.9%, *p* < 0.001) six months before the index hospital admission. This difference in prior service utilization could have influenced subsequent receipt of home-based palliative care postdischarge. However, we adjusted for prior home-based palliative care use in our final regression model examining the association between inpatient palliative care and home-based palliative care. Another difference between those who received inpatient palliative care versus those who did not is that they had lower prevalence of five or more comorbidities (24.4% vs. 34.2% *p* < 0.001). Although increasing comorbidity has been found to a play a role in access to palliative care in end of life,^45^ our analyses controlled for this variable in our final regression model.

A key finding of our study was that 5.4% of patients seen by inpatient palliative care were readmitted within 21 days of hospital discharge, before receiving any home-based palliative care, compared with 21.1% of those not seen by inpatient palliative care. A potential explanation is that patients who received inpatient palliative care were more likely to die within 21 days than those who did not. Therefore, they had less opportunity to be readmitted. Because our analyses used logistic regression rather than survival analyses, it could be that the difference in readmission rates could be partially explained by differences in survival time that were unaccounted for in our analyses.

After controlling for all covariates, our multinomial regression analysis showed the association between receiving inpatient palliative care and readmission to be insignificant. This result is in opposition to a growing body of evidence that suggests that patients who receive inpatient palliative care have lower readmissions within 30 days compared with usual care.^39,41,42,44,46^ Nonetheless, there are many mechanisms by which inpatient palliative care can reduce readmissions. A study found that compared with usual care, palliative specialists were more likely to have goal-oriented discussions with patients and to initiate new do-not-resuscitate orders.^42^ The study findings suggested that the reduction in 30-day readmission rates may be largely driven by discussions that allow the patients and providers to choose a less aggressive care plan.^42^ Discharges that result in readmissions can indicate inadequate transitional care planning or lack of comprehensive support in the community.^15,16^ At least some of the impact of inpatient palliative care on readmissions may be due to receiving community supports such as home-based palliative care.^47^

### Strengths and limitations

A major strength of this study is the use of a large population-level dataset, linked at the individual level to characterize the types of palliative care services received across the acute and home care sectors. Our results are likely generalizable to other Canadian provinces and jurisdictions that publicly fund inpatient and home-based palliative care services.

This study has several limitations. First, given that patients were not randomized to receive inpatient palliative care versus usual care, it is possible that their exposure status was influenced by their baseline characteristics. We attempted to adjust for these systematic differences between groups through controlling for demographic, morbidity, and prior utilization variables in our multivariate analysis. A limitation of using administrative health data is that we could not control for confounders such as supports available in the home, out-of-pocket care, or patient preferences for place of death. Furthermore, as we were only interested in those eligible to receive home-based palliative care, our analyses excluded decedents who were admitted or transferred to nursing homes. The exclusion criterion was applied to our study as this population was unlikely to be discharged back to the community setting where they would have received home-based palliative care. None the less, given the deficiencies in palliative care delivery in nursing homes,^14^ future research should examine the enabling factors that improve nursing home residents' access to palliative care. Finally, we did not account for the delivery of residential hospice care as a form of home-based palliative care, as it was not recorded in the health administrative databases used. However, only 1% to 3% of deaths occur in residential hospices in Ontario, and it is often initiated after delivery of home-based palliative care.^2^

## Conclusion

Health care at the end of life is predominantly concentrated in acute care. Inpatient palliative care, therefore, offers a distinct opportunity to improve transitional care between the hospital and home. Palliative care teams make efforts to encourage advanced directives, reduce aggressive medical interventions, and focus on enhancing access to comfort-based care options that may include home-based palliative care. Measuring whether inpatient palliative care impacts home-based palliative care postdischarge offers insight into the longitudinal value of inpatient palliative care. It is also one way to quantify the continuity of the palliative care approach across settings. Further research should focus on examining the types of patients who are less likely to receive palliative care as they transition across the acute and home care setting.

## Supplementary Material

Supplemental data

## Abbreviations Used

AMI: acute myocardial infarction
CHF: congestive heart failure
CI: confidence interval
CIHI-DAD: Canadian Institute for Health Information Discharge Abstract Database
COPD: chronic obstructive pulmonary disorder
OHIP: Ontario Health Insurance Plan
ORs: odds ratios
SDs: standard deviations
