# Supplementary material for: Does Inpatient Palliative Care Facilitate Home-Based Palliative Care Postdischarge? A Retrospective Cohort Study
Source: Palliat Med Rep. 2021 Feb 1;2(1):25–33. doi: 10.1089/pmr.2020.0095 (PMC8241378; doi:10.1089/pmr.2020.0095)
Supplement: Supplemental data [file Supp_Data.docx]

**S1 Table.** Description of the health administrative datasets used for this research

| Dataset | Description |
| --- | --- |
| Registered Persons Database (RPDB) | A population-based registry maintained by the Ontario Ministry of Health and Long-Term Care that contains demographic information (including age, sex, area of residence, dates of birth and where applicable, death) for all individuals who register for health insurance in Ontario |
| Canadian Institute for Health Information Discharge Abstract Database (DAD) | Contains detailed information abstracted from hospital records from all acute care centers in the province of Ontario. Psychiatric and rehabilitation admissions are contained in other health administrative datasets |
| National Ambulatory Care Reporting System (NACRS) | Includes information on hospital- and community-based ambulatory care services provided in the province of Ontario, used specifically in this study to obtain information on patient visits to emergency departments |
| Ontario Health Insurance Plan (OHIP) | Contains all claims made by all physicians for services provided to Ontario residents |
| Home Care Database | Data from the Ontario Association of Community Care Access Centers, responsible for providing all publicly funded home care |
| 2006 Canadian Census | Includes area-level markers not captured with traditional health administrative data sources (for example, area-level income) |

**S Table 2**: List of diagnostic information for defining the 17 selected chronic conditions under investigation in this study

These conditions represent a subset of all possible chronic conditions that may be experienced by individuals over a lifetime but represent the most substantial conditions from a population perspective.

| **Condition [reference for validated algorithm]** | **ICD 9 / OHIP** | **ICD 10** | **ODB*** |
| --- | --- | --- | --- |
| Acute Myocardial Infarction (AMI) [1] | 410 | I21, I22 |  |
| Osteo- and other Arthritis: |  |  |  |
| (A) Osteoarthritis | 715 | M15-M19 |  |
| (B) Other Arthritis (includes Synovitis, Fibrositis, Connective tissue disorders, Ankylosing spondylitis, Gout Traumatic arthritis, pyogenic arthritis, Joint derangement, Dupuytren’s contracture, Other MSK disorders) | 727, 729, 710, 720, 274, 716, 711, 718, 728, 739 | M00-M03, M07, M10, M11-M14, M20-M25, M30-M36, M65-M79 |  |
| Arthritis - Rheumatoid arthritis [2] | 714 | M05-M06 |  |
| Asthma [3] | 493 | J45 |  |
| (all) Cancers | 140-239 | C00-C26, C30-C44, C45-C97 |  |
| Cardiac Arrhythmia | 427 (OHIP) / 427.3 (DAD) | I48.0, I48.1 |  |
| Congestive Heart Failure [4] | 428 | I500, I501, I509 |  |
| Chronic Obstructive Pulmonary Disease [5] | 491, 492, 496 | J41, J43, J44 |  |
| Coronary syndrome (excluding AMI) | 411-414 | I20, I22-I25 |  |
| Dementia [6] | 290, 331 (OHIP) / 046.1, 290.0, 290.1, 290.2, 290.3, 290.4, 294, 331.0, 331.1, 331.5, F331.82 (DAD) | F00, F01, F02, F03, G30 | Cholinesterase Inhibitors |
| Diabetes [7] | 250 | E08 - E13 |  |
| Hypertension [8] | 401, 402, 403, 404, 405 | I10, I11, I12, I13, I15 |  |
| (Other) Mental Illnesses | 291, 292, 295, 297, 298, 299, 301, 302, 303, 304, 305, 306, 307, 313, 314, 315, 319 | F04, F050, F058, F059, F060, F061, F062, F063, F064, F07, F08, F10, F11, F12, F13, F14, F15, F16, F17, F18, F19, F20, F21, F22, F23, F24, F25, F26, F27, F28, F29, F340, F35, F36, F37, F430, F439, F453, F454, F458, F46, F47, F49, F50, F51, F52, F531, F538, F539, F54, F55, F56, F57, F58, F59, F60, F61, F62, F63, F64, F65, F66, F67, F681, F688, F69, F70, F71, F72, F73, F74, F75, F76, F77, F78, F79, F80, F81, F82, F83, F84, F85, F86, F87, F88, F89, F90, F91, F92, F931, F932, F933, F938, F939, F94, F95, F96, F97, F98 |  |
| Mood, anxiety, depression and other nonpsychotic disorders | 296, 300, 309, 311 | F30, F31, F32, F33, F34 (excl. F34.0), F38, F39, F40, F41, F42, F43.1, F43.2, F43.8, F44, F45.0, F45.1, F45.2, F48, F53.0, F68.0, F93.0, F99 |  |
| Osteoporosis | 733 | M81, M82 |  |
| Renal failure | 403, 404, 584, 585, 586, v451 | N17, N18, N19, T82.4, Z49.2, Z99.2 |  |
| Stroke (excluding transient ischemic attack) | 430, 431, 432, 434, 436 | I60-I64 |  |
| NOTES: | | | |
| Abbreviations: ICD = International Classification of Disease; ODB = Ontario Drug Benefit program database; OHIP = Ontario Health Insurance Plan, physician billings database; | | | |
| All case definitions look back to 2001 to ascertain disease status, with the exception of AMI (1 year prior to index), Cancer (2 years), Mood Disorder (2 years) and Other Mental Illnesses (2 years) | | | |
| AMI, Asthma, COPD, CHF, Dementia, Diabetes Hypertension and Rheumatoid Arthritis are based on validated case algorithms (see Sources 1-8 below, respectively). All other conditions required at least one diagnosis recorded in acute care (CIHI) or two diagnoses recorded in physician billings within a two-year period. | | | |
| *ODB prescription drug records are not available for the majority of persons under the age of 65 | | | |

**References for validated algorithms for case ascertainment**:

1. Austin PC, Daly PA, Tu JV. A multicenter study of the coding accuracy of hospital discharge administrative data for patients admitted to cardiac care units in Ontario. American Heart Journal 2002;144:290–6.
2. Widdifield J, Bernatsky S, Paterson JM, Tu K, Ng R, Thorne JC, Pope JE, Bombardier C. Accuracy of Canadian health administrative databases in identifying patients with rheumatoid arthritis: a validation study using the medical records of rheumatologists. Arthritis Care Res 2013; 65(10): 1582-1591.
3. Gershon AS, Wang C, Guan J, Vasilevska-Ristovska J, Cicutto L, To T. Identifying patients with physician-diagnosed asthma in health administrative databases. Can Respir J 2009;16:183–8.
4. Schultz SE, Rothwell DM, Chen Z, Tu K. Identifying cases of congestive heart failure from administrative data: a validation study using primary care patient records. Chronic Diseases and Injuries in Canada 2013;33:160–6.
5. Gershon AS, Wang C, Guan J, Vasilevska-Ristovska J, Cicutto L, To T. Identifying Individuals with Physician Diagnosed COPD in Health Administrative Databases. Copd 2009;6:388–94.
6. Jaakkimainen RL, Bronskill SE, Tierney MC, Herrmann N, Green D, Young J, et al. Identification of Physician-Diagnosed Alzheimer’s Disease and Related Dementias in Population-Based Administrative Data: A Validation Study Using Family Physicians’ Electronic Medical Records. J Alzheimers Dis. IOS Press; 2016 Aug 10;54(1):337–49
7. Hux JE, Ivis F, Flintoft V, Bica A. Diabetes in Ontario: Determination of prevalence and incidence using a validated administrative data algorithm. Diabetes Care 2002;25:512–6.
8. Tu K, Campbell NR, Chen Z-L, Cauch-Dudek KJ, McAlister FA. Accuracy of administrative databases in identifying patients with hypertension. Open Med 2007;1:e18–26.

**S3 Table.** Counts per study inclusion criterion

| **Step** | **Description** | **# Excluded** | **Total Cohort remaining** |
| --- | --- | --- | --- |
| 1 | Decedents 2013 to 2016 in RPDB | Before Exclusion | 287,154 |
| 2 | Age<19y or age>105y at death | 3,926 | 283,228 |
| 3 | Non-Ontario postal code at death | 1,738 | 281,490 |
| 4 | Among IKNs all acute hospital discharges in last 6months |  | 245,047 |
| 5 | Data quality issues at death date | 156 | 244,891 |
| 6 | Long-term care flag in last 6months | 85,311 | 196,179 |
| 7 | OHIP ineligible at any point in last 1y | 788 | 195,391 |
| 8 | Data quality issues in last 6months | 1,072 | 194,319 |
| 9 | Discharge disposition not to community | 125,088 | 119,803 |
| 10 | Discharge not within 30 days of death | 30,381 | 89,422 |
| 11 | First discharge | 30,414 | 59,008 |
